# Roles for non-human primate-associated phage diversity in improving medicine and public health

**DOI:** 10.1093/emph/eoac006

**Published:** 2022-02-18

**Authors:** Jan F Gogarten

**Affiliations:** 1 Applied Zoology and Nature Conservation, University of Greifswald, Loitzer Str. 26 17489 Greifswald, Germany; 2 Epidemiology of Highly Pathogenic Microorganisms, Robert Koch Institute, Berlin, Germany; 3 Viral Evolution, Robert Koch Institute, Berlin, Germany

**Keywords:** wildlife, phage therapy, bacteriophage, primates, emerging infectious disease risk

## Abstract

Mammals harbor trillions of microorganisms and understanding the ecological and evolutionary processes structuring these ecosystems may provide insights relevant to public health and medicine. Comparative studies with our closest living relatives, non-human primates, have provided first insights into their rich bacteriophage communities. Here, I discuss how this phage diversity can be useful for combatting antibiotic-resistant infections and understanding disease emergence risk. For example, some primate-associated phages show a pattern suggesting a long-term co-divergence with their primate superhosts—co-diverging phages may be more likely to exhibit a narrow host range and thus less useful for phage therapy. Captive primates lose their natural phageome, which is replaced by human-associated phages making phages an exciting tool for studying rates of microorganism transmission at human–wildlife interfaces. This commentary tackles avenues for selecting phages for therapeutic interventions based on their ecological and evolutionary history, while discussing frameworks to allow primate-associated phages to be incorporated into the arsenal of clinicians.

## Lay Summary

Evidence suggests that mammals harbor trillions of microorganisms on and in their bodies. Understanding the ecological and evolutionary processes structuring these ecosystems has the potential to provide insights relevant to public health and medicine, some of which are explored here. Specifically, I examine ways in which the phage diversity of non-human primates could be useful for combatting antibiotic-resistant infections and understanding disease emergence risk. This includes an exploration of ways to select phages for therapeutic interventions based on their ecological and evolutionary history, as well as how phages might be useful for studying rates of microorganism transmission at human–wildlife interfaces. The hope is that these frameworks may allow primate-associated phages to be incorporated into the arsenal of clinicians.

## INTRODUCTION

Every mammal hosts trillions of microorganisms; humans are no exception. These communities constitute a majority of the cells found in a host and include bacteria, archaea, microbial eukaryotes as well as many viruses infecting these cellular organisms (including bacteria-infecting bacteriophages; hereafter phages; Box 1) [1]. Beyond clear instances of bacteria or viruses impacting a host’s health as pathogens, these microbial communities impact a broad range of processes including a host’s ability to access nutrients [2] and health via pathogen exclusion and immune system priming [3]. The most abundant and diverse communities are found in the mammalian gut [1] and there, community composition is related to many human diseases [4, 5] and long-term mortality risk [6]. Inspired by these insights, researchers increasingly consider the human body as an ecosystem, with human health a key product of the ecosystem services generated by their microbial communities [7]. Pathogens can invade these ecosystems and cause detrimental impacts on host health, but a dysbiosis (i.e. imbalance in their composition and functional capabilities) in these communities can have a similar negative impact on host health. Ecological and evolutionary processes play a major role in structuring these ecosystems and the services they provide, and understanding these processes can provide insights relevant to both public health and medicine [8].

Gut bacterial communities host diverse phage communities with the potential to influence their structure, function, colonization patterns, and ultimately, superhost health (a superhost hosts the bacteria that host the phage) [9]. Indeed, enriched phage communities in human intestinal mucus act as an acquired immune system by limiting mucosal bacterial populations [10], while dysbiotic gut phageomes are associated with diseases such as Type II diabetes [5], colitis [4] and stunting [11]. Transplantation of healthy viral filtrates restored health in patients infected with *Clostridium difficile* [12], while *in vitro* studies suggest phages from stunted children shape bacterial populations differently than those of healthy children [11], supporting a direct link between phageome composition and disease and raising the prospect of using phages as therapeutics. While components of the healthy human phageome composition appear widely distributed across the human population, individuals often appear to have distinct phageomes [13]. Some phages remain stable members of an individual’s phageome for at least 26 months, suggesting these populations are not subject to dramatic periodic fluctuations and classical ‘kill the winner dynamics’ and are rather long-term members of this ecosystem [13]. Despite their importance, the ecological and evolutionary processes that gave rise to these phage communities in humans are only beginning to be unraveled.

## FIRST INSIGHTS INTO THE ORIGINS OF THE HUMAN PHAGEOME

Comparative studies with our closest living relatives, non-human primates, can provide insights into the evolutionary and ecological processes shaping our microbial communities [14]. The discovery of crAssphage (cross-assembly phages) relatives in a diversity of non-human primates was a first hint that phages might have maintained long-term associations with their superhosts across millions of years of evolutionary history, a pattern similar to what has been described for some bacteria [15]. This finding was the inspiration for a recent study using a database of healthy human-associated phages as an anchor to explore the ecological drivers of phage community assembly and individual phage lineage evolution in primate superhosts across multiple scales and environments [16]. It was possible to identify phages in fecal shotgun metagenomes from 23 wild non-human primate taxa, as well as from humans living in Africa and Germany, and from four captive great ape taxa and their zookeepers. More than that, 60% of human-associated phages had relatives in at least one wild non-human primate superhost taxon and nearly 30% had relatives in five or more non-human primate taxa [16]. Furthermore, primate taxa had distinct phageome compositions that exhibited a clear phylosymbiotic signal, with more closely related species harboring more similar phage community compositions. The finding of shared phages could reflect the presence of generalist phages that are shared between superhosts, rather than a long-term association which would be reflected in patterns of co-divergence. Interestingly though, this phylosymbiotic signal appeared to be driven in part by phage–superhost co-divergence; a pattern observed in 44 individual phage lineages [16]. Some bacteria also appear to exhibit a pattern of co-divergence with their hosts [15, 17] and by linking phages to their particular bacterial hosts, it may be possible to determine whether co-diverging phages tend to infect co-diverging bacteria.

The support for the hypothesis of long-term co-divergence between phages and superhosts raises the question of how such long-term co-divergence over millions of years is possible, when many distantly related species of primates are sympatric, living in the same ecosystem? The study of phages within a single primate species, baboons, revealed that neighboring social groups harbored evolutionarily and compositionally distinct phageomes that were structured by their superhost’s social behavior; close grooming partners had more similar phage communities, even after controlling for relatedness or diet of the superhosts [16]. This study joined a growing body of literature suggesting that maintenance of microbial communities within groups through social behavior serves to create a pan-microbiome that can act to maintain microbial diversity across evolutionary time scales and contributing to the evolution of host species-specific gut microbial communities [18, 19].

Surprisingly, a first study of the captive primate phageome found that captive non-human primate phageomes were more similar to humans than their wild counterparts, with nearly complete replacement of wild-associated phages with human-associated ones. This finding was then also found for bacteria, where patterns of co-divergence with their primate hosts was observed in the wild [15], but subsequently absent for captive primates [17]. This suggests that primate–phage associations can be extremely labile in captivity [16]. This finding makes the fact that in the wild, relationships between some phages and their superhosts appear to have persisted across millions of years, potentially facilitated by transmission between groupmates, all the more striking.

Broadly speaking, this preliminary body of work suggests that humans have distinct phageome communities when compared to those of their ancestors; understanding how changes in these communities over recent evolutionary history have contributed to the rise of disease of gut inflammation and health more broadly is a key area of future research. A key limitation of this study was the limited sampling of the human population (*n*_Democratic Republic of Congo_, *n*_Côte d’Ivoire_ = 12, *n*_Germany_ = 24), as well as across non-human primate populations. Studies of the bacterial microbiome suggest major differences between industrialized and hunter-gather societies [20, 21], as well as between different non-human primate populations [22]. A broader study of human and non-human primates under different ecological contexts will help generate a more complete understanding of the diversity of their phages and the factors that influence these communities. Recent mining of available human shotgun metagenomes suggests many phages are widely distributed across human populations [23]. Despite their significance, these preliminary studies of the non-human primate phageome were also limited by their use of the human-associated phageome as an anchor for detecting phages [16]. A critical question for the field to explore is the broader diversity of primate phages to address whether humans have lost phageome diversity during their recent evolutionary history.

While non-human primates represents one insight into the history of our associations with phages and other viruses, ancient DNA studies of human coprolites are also shedding light onto the diversity of phages that were historically harbored in the human gut [24]. Combining such archeovirological sources of data on the human phageome with information on the phages found in association with non-human primates is likely to provide the clearest perspective into potential changes in these associations that occurred during major changes in how humans live (e.g. the first epidemiologic transition associated with the Neolithic) [25]. Below, I explore how insights from such studies could inform medicine and public health.

## PHAGE THERAPY

The discovery of antibiotics greatly improved our ability to cope with bacterial infections, increasing human health and agriculture efficiency [26]. While antibiotics represent useful tools, antibiotic resistance occurs when bacteria evolve to resist these compounds and most antibiotics are broad-spectrum and can have unintended consequences on the broader symbiotic host microbiome. The overuse of antibiotic compounds also exerts a strong pressure for the evolution of resistance and once this arises, it can spread widely through the selective advantages of particular strains and between bacterial species through horizontal gene transfer [26]. Indeed, resistance to all major groups of antibiotics used by humans arose quickly after their development [27]. Antibiotic resistance has emerged as a major threat to public health that is predicted to have a massive human and economic impact. The World Health Organization estimates that antimicrobial resistance causes 700 000 deaths annually and that microbes resistant to all available antimicrobial treatments may kill up to 10 million people annually by 2050 [27]. If no action is taken, the economic impact is predicted to be profound too. Lost global production between by 2050 is estimated to be US$100 trillion [28]. Given this bleak prognosis, the development of new and effective antibiotic therapies is critical.

This rise of multi-drug resistant bacterial infections led to a resurgence of interest in phage therapy. Interest is typically in phages that self-amplify and kill the bacteria. These lytic phages attach to a bacterium, inject its genome into the host, and use the host’s cellular machinery to make copies of itself, ultimately lysing the cell and releasing progeny in search of more hosts, making them ideal for the treatment of bacterial infections. After the discovery of lytic phages in 1915, their use in treatment of bacterial infections was actively explored [29]. Phages typically infect a narrow range of bacteria and are not able to infect human cells. This host specificity and the fact that phages are produced in areas of the body where a bacterial infection is active with production decaying when the population of bacteria has been destroyed, make them a promising therapeutic agent. Despite early successes, the ease of antibiotic production and administration, coupled with the fact that phage therapy gained early popularity in Eastern Europe and became associated with communism, contributed to phages not being widely pursued as therapeutic agents in Western Europe and the Americas. This contributed to a lack of clinical trials, infrastructure and regulation for phage production, which limited their use [though these hurdles are beginning to be overcome: 30].

Phages are found everywhere bacteria exist and new technology enables insights into this diversity [30]. A critical task for phage therapy is the selection of virulent and effective phages for bacteria that cause human disease [31]. Classically, bacterial susceptibility to antibiotics has been accessed by culturing bacteria with different antibiotics and a similar approach can be used with phages, mixing a phage-containing sample with host bacteria and searching for evidence of lysis [31]. While a narrow phage host range is useful in that the phage will not influence the symbiotic microbiome, phages can be specific to a particular strain, complicating their use in therapeutic interventions [32]. Thus, a broader host range is typically considered desirable for phage therapy in that it may reduce treatment failures due to mismatching of a phage to the particular strain causing an infection [32]. In addition, the use of multiple phages in a cocktail can essentially increase the effective host range of a treatment and as resistance to phages evolves as well, the use of multiple phages in combination can reduce the probability of bacteria being resistant to all of them [33].

This suggests that increasing the number of phages available to target a bacterial infection will be beneficial for phage therapy, as is finding phages with a relatively broad host range. National and international efforts are underway to create resources (i.e. phage banks) for the cataloguing of phages for their rapid deployment in clinical settings. To date, there is a limited availability of multiple broad host range phages for inclusion in phage cocktails to test on emerging antimicrobial resistant bacteria [34]. Bacterial isolates that are not covered by these phage banks are thus used as targets for new phage discovery to expand the phage toolbox that could be challenged by novel sources of phages.

## PRIMATE-ASSOCIATED PHAGEOMES AS SOURCES OF THERAPEUTIC PHAGES

One particularly common source of phages for such challenge experiments is human sewage or wastewater [35]. Comparisons of the human gut microbiome with that of our closest living relatives suggest that humans, particularly those in industrialized nations, harbor far fewer gut bacterial taxa than any other primate [36]. Not only do the depauperate microbiomes of humans potentially predispose us to a variety of diseases, it also suggests human feces may not be a particularly rich source of phage diversity for phage therapy. Primate guts contain a multitude of phages that infect a similar range of bacteria to those found in humans, but they may also harbor phage diversity lost to the human lineage. This diversity may represent an untapped resource for phage therapy, particularly for the identification of phages with a broader host range that are still relevant to the bacteria infecting humans.

These phage communities act as modulators of the gut microbiota and the extant non-human primate phageome could also provide phages that are useful for therapeutic interventions to modify a dysbiotic gut microbiome. Indeed, it is often hypothesized that many different diseases associated with industrialized nations are associated with our shrinking microbiomes [36]. The use of whole virome transplants to counter gut dysbiosis is showing promise, but faces challenges (reviewed in Ref. [37]). The non-human primate phageome likely impacts human gut microbiomes quite differently than the phageome of modern humans, and represents an alternative source of whole viromes for therapeutics that could be first explored in animal models.

Captivity modifies both the bacterial and phage communities found in the non-human primate gut [16, 38]. This means that efforts to harness non-human primate gut phage diversity will have to sample wild primates. Primatologists often collect fecal samples to study aspects of their subjects’ lives (e.g. diet [39], eukaryotic parasites [40]), meaning that large collections of samples exist that could be used for such efforts. Surprisingly, it is possible to culture the majority of bacterial genera found in the vertebrate gut microbiota from frozen fecal samples, even those stored in RNAlater [41]. The phages infecting these bacteria are likely also viable [42], which enables access to the phages in vast collections of fecal samples and make isolating wild primate-associated phages a feasible task. For example, phage-containing primate feces could be mixed with human pathogens to expand the search for lytic phages. At the same time, long-term observational studies of wild primates, like the Amboseli Baboon Research Project, provide additional metadata that allow researchers to address questions that are quite difficult to address in humans. These baboons have been followed for decades, with researchers collecting data on their genealogy, diet and grooming partners, coupled with frequent fecal sampling; such a resolution of diverse sources of data coupled with regular sampling does not exist for human populations, providing many new insights [43].

A promising avenue for selecting phages for therapeutic interventions is the incorporation of data on the ecological and evolutionary history of these phages across the primate lineage. Phages that have a history of superhost switching within ecosystems are very likely to have a broader bacterial host range [16], making them promising therapeutic agents. In contrast, phages with a strong signal pointing toward superhost specificity over evolutionary timescales, despite their primate superhosts sharing an ecosystem, might be less likely to have a broad host range, and thus less useful tools for phage therapy.

Because of their immunological and physiological similarities, non-human primates are infected by many closely related pathogenic bacteria. Indeed, the phylogenetic proximity of two organisms predicts the likelihood of pathogen jumps and many human pathogens have relatively recent origins in non-human primate populations [14]. This suggests that many bacterial pathogens, for which phage therapy might represent a useful intervention, might be recent arrivals in the human population. The field of invasion biology suggests that when an organism invades a new area, they often escape the parasites found in their historic range [44]. Similar dynamics may occur when bacterial pathogens emerge in the human population (i.e. akin to a species invading a new area). Bacteria may have left behind the phages that limited their populations in their historic hosts. If this is the case, the phage diversity of bacterial pathogens in wildlife hosts could be particularly useful for treating human infections. To get access to such phages, samples could be collected from an animal infected with a particular bacterium of interest. Some health monitoring projects of primates, such as the Taï Chimpanzee Project in the Côte d’Ivoire, have a veterinarian in the field who collects necropsy samples using rigorous biosafety procedures [45]. This sampling has yielded insights into a broad range of bacteria that infect primates, though the phages infecting these bacteria have, to date, not been systematically examined. In other instances, symptomatic primates are anesthetized in the field, allowing high-quality sample collection from lesions [46]. A rich collection of necropsy samples and samples from symptomatic individuals has the potential to serve as a rich resource for the isolation of phages useful for therapeutic interventions against antimicrobial resistant bacteria infecting humans.

## PRIMATE-ASSOCIATED PHAGES FOR PUBLIC HEALTH AND DISEASE RISK ASSESSMENTS

The transmission of pathogens occurs not only from wildlife to humans, but also from humans to wildlife, and can pose a risk to endangered populations [47]. Understanding human–primate microorganism transmission in areas where regular contact occurs, as well as what can be done to mitigate this transmission, represents a cornerstone for minimizing the global risk of novel disease emergence and for wildlife conservation. Detecting transmission events as they happen requires health monitoring of both wildlife and human populations. The rarity of these transmission events generally prevents their direct observation. In a few cases, scientists can provide insights into a potential wildlife species that was the source of a human outbreak by mobilizing rapidly at the start of a human epidemic to sample neighboring wildlife in those locations. Often though, sampling of potential reservoir species occurs well after a spillover event. While challenging, such data can still generate information that enables the identification of potential sources of a pathogen and insights into the context in which it occurred. Ultimately, our understanding of factors facilitating disease emergence is largely predicated on research detecting historic transmission by looking at the evolutionary relatedness of human and wildlife microorganisms [14, 25].

Phages represent an exciting tool to study rates of microorganism transmission at the human–wildlife interface. This is in part because research suggests phages have faster life histories and evolutionary rates than bacteria [48], meaning their genomes quickly accumulate variation that can be used to infer their evolutionary histories. In addition, in contrast to eukaryotic viruses that must overcome significant hurdles to adapt to a new host, significant overlap in the bacterial communities found in humans and primates suggests suitable bacterial hosts may often be present in human and primate guts. This may make phage transmission between superhosts much more frequent than the transmission of eukaryotic viruses. Indeed, studies of primate-associated phages of captive primates demonstrated high rates of transmission from humans to great apes [16]. In addition, diverse phages represent a large part of the nucleic acid found in feces, meaning one is not looking for a needle in the haystack. Phages thus may provide a toolkit to test whether intervention efforts are successful in reducing the potential for spillover of other more deadly pathogens, as well for generating and understanding of the factors that increase spillover risk. Despite this potential, the small genomes and high mutation rates that characterize many phages may pose challenges for efforts to apply population genetic models and the accurate estimation of phylogenetic timescales. For example, a loss of phylogenetic information may occur due to substitution saturation, particularly for deep branches and testing for substitution saturation may be prudent for distantly related phages [49]. Despite these challenges, the reconstruction of vertebrate infecting RNA virus using virus replicases suggests that the deep evolutionary structure of fast evolving viruses is possible for the time scales of interest when considering primate-associated phages [50].

## OUTLOOK

**Figure 1.**
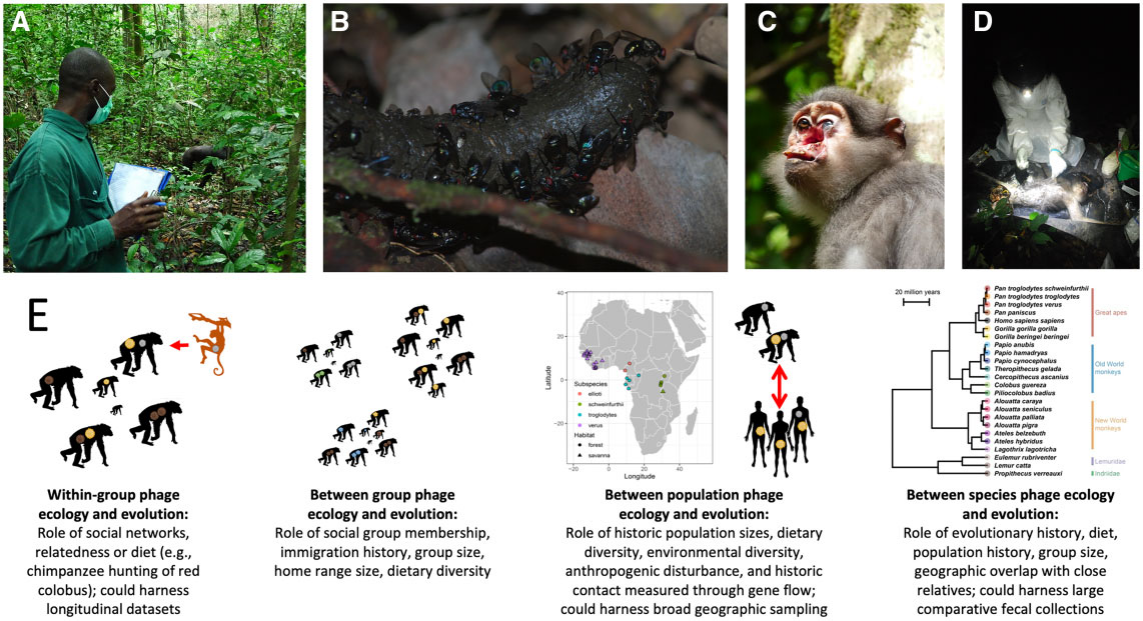
(**A**) Primatologists and veterinarians working with wild non-human primate populations can collect primate-associated phages from: (**B**) fecal samples, (**C**) swabs taken from sick animals like this mangabey infected with *Treponema pallidum pertenue* or (**D**) necropsy samples taken from a carcass when an animal dies. (**E**) These samples can then provide data to study the ecological and evolutionary processes shaping the primate-associated phageome on a number of scales

Understanding the ecological and evolutionary factors that shaped the diversity of phages found in humans and our closest living relatives may pave the way for harnessing this diversity for medical interventions like phage therapy. Implementing such interventions with these primate-associated phages will require significantly more research and coordination with regulators and clinicians, which may be best achieved by contributing to phage banks [30, 32, 34]. Phages also represent an exciting tool to study rates of microorganism transmission at the human–wildlife interface that may help predict disease emergence and help target interventions. New technologies coupled with extensive environmental sampling will allow the study of primate-associated phage communities on a number of scales (Fig. 1). The development of scalable and cost-effective tools to conduct more temporally and spatially broad analyses (e.g. phage family metabarcoding systems) will be helpful for maximizing the efficiency of such efforts. Alignments of phage genomes from across non-human primates represent a promising approach for the development of such systems [16]. Such a toolkit would ultimately enable a detailed examination of primate phage ecology, in the primate’s natural social and environmental context, but also for those aiming to monitor phage dysbiosis in human health conditions.
